# Sauchinone Ameliorates Senescence Through Reducing Mitochondrial ROS Production

**DOI:** 10.3390/antiox14030259

**Published:** 2025-02-24

**Authors:** Myeong Uk Kuk, Yun Haeng Lee, Duyeol Kim, Kyeong Seon Lee, Ji Ho Park, Jee Hee Yoon, Yoo Jin Lee, Byeonghyeon So, Minseon Kim, Hyung Wook Kwon, Youngjoo Byun, Ki Yong Lee, Joon Tae Park

**Affiliations:** 1 Division of Life Sciences, College of Life Sciences and Bioengineering, Incheon National University, Incheon 22012, Republic of Korea; muk20@inu.ac.kr (M.U.K.); yh.lee@inu.ac.kr (Y.H.L.); papaya1130@inu.ac.kr (D.K.); 202428002@inu.ac.kr (J.H.P.); yoojn0905@inu.ac.kr (J.H.Y.); juli9709@inu.ac.kr (Y.J.L.); tundra@inu.ac.kr (B.S.); alstjs0323@inu.ac.kr (M.K.); hwkwon@inu.ac.kr (H.W.K.); 2 College of Pharmacy, Korea University, Sejong 30019, Republic of Korea; kslee0118@korea.ar.kr (K.S.L.); yjbyun1@korea.ac.kr (Y.B.); 3 Interdisciplinary Major Program in Innovative Pharmaceutical Sciences, Korea University, Sejong 30019, Republic of Korea; 4 Convergence Research Center for Insect Vectors, Incheon National University, Incheon 22012, Republic of Korea

**Keywords:** mitochondrial oxidative stress, mitochondria, ROS, senescence amelioration

## Abstract

One of the major causes of senescence is oxidative stress caused by ROS, which is mainly generated from dysfunctional mitochondria. Strategies to limit mitochondrial ROS production are considered important for reversing senescence, but effective approaches to reduce them have not yet been developed. In this study, we screened the secondary metabolites that plants produce under oxidative stress and discovered sauchinone as a potential candidate. Sauchinone induced mitochondrial function recovery, enabling efficient electron transport within the electron transport chain (ETC). This led to a decrease in ROS production, a byproduct of inefficient electron transport. The reduction in ROS by sauchinone rejuvenated senescence-associated phenotypes. To understand the underlying mechanism by which sauchinone rejuvenates senescence, we carried out RNA sequencing and found *VAMP8* as a key gene. *VAMP8* was downregulated by sauchinone. Knockdown of *VAMP8* decreased mitochondrial ROS levels and subsequently rejuvenated mitochondrial function, which was similar to the effect of sauchinone. Taken together, these studies revealed a novel mechanism by which sauchinone reduces mitochondrial ROS production by regulating mitochondrial function and *VAMP8* expression. Our results open a new avenue for aging research to control senescence by regulating mitochondrial ROS production.

## 1. Introduction

Senescence is defined as the loss of replicative capacity of normal somatic cells after prolonged division or due to various stresses, such as oxidative stress, telomere dysfunction, and DNA damage [1,2]. The hallmarks of senescence are cell cycle arrest, telomere erosion, lipofuscin accumulation, senescence-associated β-galactosidase activity, larger cell shape, and senescence-associated secretory phenotypes [3]. Senescence is also characterized by deteriorations in the structure and function of cellular organelles [4]. Among these organelles, the mitochondria exhibit structural changes that increase in size and volume, resulting in dysfunctional mitochondria [5]. Dysfunctional mitochondria leak electrons in the electron transport complex (ETC), generating reactive oxygen species (ROS) [6]. To make up for mitochondrial dysfunction, ROS triggers a feedback response that increases mitochondrial size and mass.

ROS are oxygen-containing chemically reactive molecules, which include nonradical species like hydrogen peroxide (H2O₂) and free radicals like hydroxyl radical (^●^OH) and superoxide anion (O₂⁻) [7,8,9]. Under normal physiological conditions, ROS play essential roles in homeostasis, cell signaling, and defense against pathogens [10,11,12]. However, excessive ROS above physiological levels cause oxidative stress, which harms cellular constituents [13,14,15]. Dysfunctional mitochondria are not only a major source of ROS production but are also the organelles directly damaged by ROS [16]. Excessive ROS cause more mitochondrial damage, which increases the generation of ROS within the mitochondria. The oxidative stress theory of senescence posits that the oxidative damage caused by ROS is a major cause of senescence [17,18,19]. Considering that oxidative stress impairs tissue and organ functions [20,21,22], modulating ROS levels has become an important strategy in anti-aging research [23,24,25]. Despite the recognition of the importance of ROS regulation, strategies to prevent senescence by lowering ROS levels are still lacking, and continued research in this area is needed.

Secondary metabolites are produced by plants and are divided into three major groups: terpenes, phenols, and nitrogen-containing compounds [26]. Secondary metabolites are essential for various physiological functions, including pathogen defense and stress responses [26]. The protective activities of secondary metabolites are related to free-radical scavenging, antioxidant, and chelating activities [27]. Pinusolide, a terpene compound extracted from pine extracts, has potent antioxidant and anti-inflammatory activities [28]. Sauchinone, a low-molecular-weight polyphenol, is known for its anti-inflammatory properties [29]. Puerarin, an isoflavone belonging to polyphenols, has potent antioxidant and anti-inflammatory effects [30]. Despite their promising outcomes, the underlying mechanisms of these secondary metabolites are still unclear and need more research.

In this study, we identified that sauchinone significantly reduces mitochondrial ROS production in senescent fibroblasts. Sauchinone restored senescence-related phenotypes through a novel ROS-reducing mechanism. Furthermore, transcriptome analysis confirmed the underlying mechanism of sauchinone-induced senescence amelioration. Here, we propose sauchinone-based mitochondrial ROS reduction as an important tool for treating senescence.

## 2. Materials and Methods

### 2.1. Cell Culture

In this study, human dermal fibroblasts (PCS-201-010; ATCC, Manassas, VA, USA) and human embryonic kidney cells (HEK293T; CRL-11268; ATCC) were used. Each cell was cultured according to a previous study method [31]. Human dermal fibroblasts were classified as either senescent or young if their doubling time was greater than 14 days or less than 2 days, respectively.

### 2.2. Preparation of Compound Library

Pinusolide, sauchinone, and puerarin were isolated from *Biota orientalis*, *Saururus chinensis*, and *Pueraria lobata*, respectively [32]. Pinusolide’s structure was determined using ^1^H–nuclear magnetic resonance (NMR) and ^13^C–NMR (Figures S1 and S2). Puerarin’s structure was determined using ^1^H–NMR and ^13^C–NMR (Figures S3 and S4). Sauchinone’s structure was determined using ^1^H–NMR, ^13^C–NMR, electrospray ionization mass spectrometry, and high-performance liquid chromatography (Figures S5–S8). Pinusolide, sauchinone, or puerarin were diluted to 40 mM with dimethyl sulfoxide (DMSO, D8418; Sigma, St. Louis, MO, USA). Ten mL of medium was combined with 1 μL of 40 mM pinusolide, sauchinone, or puerarin each to reach a concentration of 4 μM. By diluting DMSO to a concentration of 0.01% in the medium, DMSO control was employed.

### 2.3. Flow Cytometric Analysis of Reactive Oxygen Species (ROS)

Senescent fibroblasts were administered with DMSO (0.01%), pinusolide (4 µM), sauchinone (4 µM), or puerarin (4 µM) for 12 days. Then, the cells were administered with a medium containing 30 µM dihydrorhodamine 123 (DHR123; 10056-1; Biotium, Fremont, CA, USA) for 30 min at 37 °C to quantify the mitochondrial ROS levels [31].

### 2.4. Cell Proliferation Assay

Cells were seeded at 1 × 10^3^ per well in 96-well plates (353072; Corning, Corning, NY, USA). DMSO (0.01%) or sauchinone (0.25, 0.5, 1, 2, or 4 µM) was administered to senescent fibroblasts at 4-day intervals for 12 days. Young fibroblasts served as a positive control. EZ-cytox reagent (EZ-5000; DoGenBio, Seoul, Republic of Korea) was used for cell proliferation. It was based on the 3-[4,5-dimethylthiazol-2-yl]-2,5 diphenyl tetrazolium bromide (MTT) assay. After diluting 1:10 in the cell culture medium, 100 µL of the diluted EZ-cytox were added to each well. Plates were incubated at 37 °C for 1 h in a 5% CO_2_ incubator. After incubation, absorbance was measured at 460 nm using a VICTOR Multilabel Plate Reader (2030-0050; PerkinElmer, Waltham, MA, USA).

### 2.5. Determination of Cell Viability

Senescent fibroblasts were administered with DMSO (0.01%) or sauchinone (1 μM) at 4-day intervals for 12 days. Cell viability was measured by trypan blue staining using a Cedex HiRes analyzer (05650216001; Roche, Basel, Switzerland) [33]. Brightfield cell images were automatically captured on the Cedex HiRes analyzer. Cell viability was measured using digital image recognition technology in the Cedex HiRes analyzer [33].

### 2.6. Flow Cytometric Analysis of Mitochondrial Membrane Potential (MMP), Mitochondrial Mass, Lysosomal Mass and Autofluorescence

Senescent fibroblasts were administered with DMSO (0.01%) or sauchinone (1 µM) for 12 days. Young fibroblasts served as a positive control. To assess MMP, cells were stained in a medium containing 0.6 µg/mL JC–10 (ENZ–52305; Enzo Life Sciences, Farmingdale, NY, USA) for 30 min at 37 °C. To assess mitochondrial mass, cells were stained in a medium containing 50 nM MitoTracker™ Deep Red FM Dye (M46753; Invitrogen, Waltham, MA, USA) for 30 min at 37 °C. To assess lysosomal mass, cells were stained in a medium containing 500 nM LysoTracker™ Green DND–26 (LTDR) (L7526; Thermofisher Scientific, Waltham, MA, USA) for 30 min at 37 °C. To assess autofluorescence, cells were stained in a dye-free medium at 37 °C for 30 min. FACS analysis was then performed as previously described [34].

### 2.7. Analysis of the Extracellular Acidification Rate (ECAR)

The Seahorse XFe96 analyzer (Aglient Technologies, Santa Clara, CA, USA) was used in accordance with product instructions. As previously stated, ECAR was performed using the Seahorse XF Glycolytic Rate Assay Kit (103344-100; Aglient Technology) [35].

### 2.8. Immunofluorescence

The immunofluorescence was performed following previously published methodology [35]. The primary antibodies were LC3B (anti-rabbit) (A19665; 1:500 dilution; Abclonal, Boston, MA, USA) and Total OXPHOS Human WB Antibody Cocktail (anti-mouse) (ab110411; 1:200 dilution; Abcam, Cambridge, UK). The secondary antibodies were Alexa Fluor^®^ 647 goat anti-mouse IgG antibody (A-28181; 1:200 dilution; Invitrogen) and Alexa Fluor^®^ 488 goat anti-rabbit IgG antibody (A-11008; 1:200 dilution; Invitrogen). Images were captured using a Carl Zeiss LSM 700 confocal microscope (Zeiss, Oberkochen, Germany).

### 2.9. Analysis and Quantification of Autophagy Flux

Senescent fibroblasts were administered with DMSO (0.01%) or sauchinone (1 µM) for 12 days. Young fibroblasts served as a positive control. Twenty-four h before flow cytometric analysis, the cells were administered with (w/) or without (w/o) 20 μM chloroquine (CQ). Then, the cells were stained for 30 min with Cyto-ID staining solution (ENZ-51031-0050; Enzo Life Sciences) or 500 nM LTDR (L7526; Thermofisher Scientific). Mean fluorescence intensity is MFI. Autophagic flux = [MFI Cyto-ID (w/ CQ)/MFI LTDR (w/ CQ)] − [MFI Cyto-ID (w/o CQ)/MFI LTDR (w/o CQ)].

### 2.10. Neutral Comet Assay

DNA tail length was determined using the CometAssay Single Cell Gel Electrophoresis Assay Kit (4250-050-K; R&D Systems, Minneapolis, MN, USA) according to the manufacturer’s instructions. DNA tail length was measured using Image j (National Institute of Health, Bethesda, MD, USA).

### 2.11. Quantitative Polymerase Chain Reaction (qPCR)

A qPCR was carried out as previously described [35]. The primers used for qPCR are provided in Table 1.

### 2.12. Transcriptome Expression Profiling

Total RNA was collected to profile transcriptome expression in senescent fibroblasts administered with DMSO (0.01%) or sauchinone (1 μM) for 12 days. Total RNA was generated using the RNase Mini Kit (74104; QIAGEN, Hilden, Germany) following the manufacturer’s instructions. A comprehensive RNA-sequencing approach was followed according to the procedures described in a previous study [36]. Three biological replicates of transcripts were used for expression analysis.

### 2.13. Preparation of shRNA

pLKO.1-puro lentiviral vector (8453; Addgene, Watertown, MA, USA) was used to subclone *VAMP8* shRNA. Table 2 lists the oligomers used to produce *VAMP8* shRNA (1), *VAMP8* shRNA (2), and *VAMP8* shRNA (3). To make the *VAMP8* shRNA mixture, *VAMP8* shRNA (1), *VAMP8* shRNA (2), and *VAMP8* shRNA (3) were combined in a 1:1:1 ratio. A pLKO.1-puro lentiviral vector was used for control shRNA.

### 2.14. Lenti–Viral Production and Infection

Using Lipofectamine 2000 (11668019; Invitrogen), 5 μg shRNA plasmid (control shRNA or VAMP8 shRNA combination), 2.5 μg PAX2 plasmid, and 2.5 μg VSV–G plasmid were transfected into HEK 293T cells. Twenty-four hours after transfection, viral supernatants were gathered. Polybrene (TR–1003–G; 6 μg/mL; Millipore, Burlington, MA, USA) was added to viral supernatants. As previously reported, viral infections were carried out [35].

### 2.15. Statistical Analysis

The statistical analyses were performed using GraphPad Prism 9 (San Diego, CA, USA). Student’s *t*-test and two-way ANOVA followed by Bonferroni’s post hoc test were used.

## 3. Results

### 3.1. Sauchinone Reduces Mitochondrial ROS Levels in Senescent Fibroblasts

**Figure 1:**
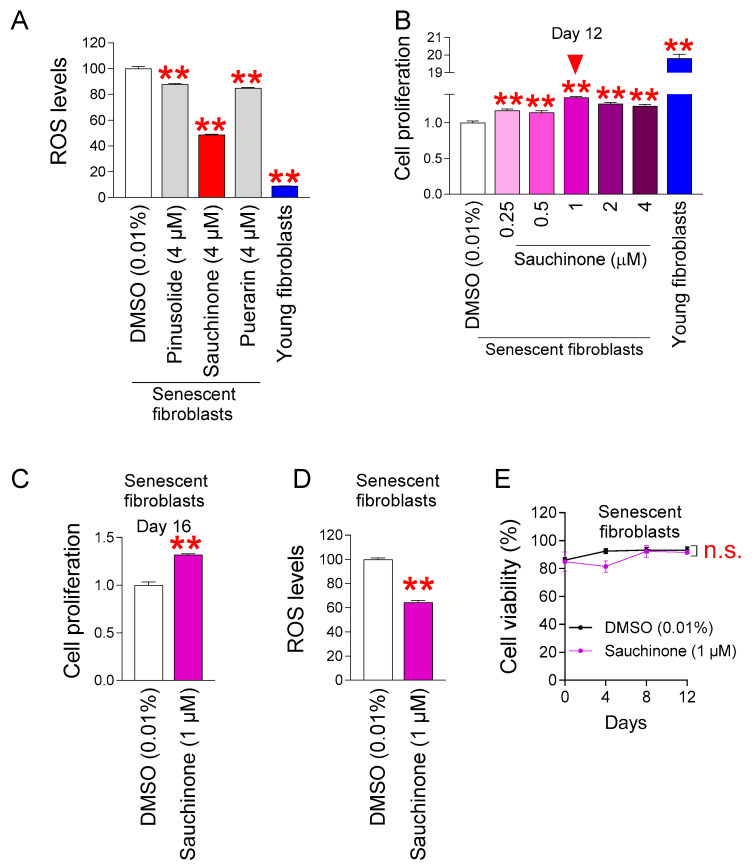
Sauchinone reduces mitochondrial ROS levels in senescent fibroblasts. (**A**) Senescent fibroblasts were treated with DMSO (0.01%), pinusolide (4 μM), sauchinone (4 μM), or puerarin (4 μM) for 12 days. Young fibroblasts served as a positive control. Use of dihydrorhodamine 123 (DHR123) to detect mitochondrial ROS levels. Statistical analysis was performed using Student’s *t*-test, with results considered significant at ** *p* < 0.01. Data represent the mean ± S.D., *n* = 3. (**B**) Senescent fibroblasts were administered with DMSO (0.01%) or different concentrations of sauchinone (0.25, 0.5, 1, 2, and 4 µM) for 12 days. Then, cellular proliferation based on MTT assay was evaluated. Young fibroblasts served as a positive control. Statistical analysis was performed using Student’s *t*-test, with results considered significant at ** *p* < 0.01. Data represent the mean ± S.D., *n* = 3. (**C**) Senescent fibroblasts were administered with DMSO (0.01%) or sauchinone (1 µM) for 16 days. Then, cellular proliferation based on MTT assay was evaluated. Statistical analysis was performed using Student’s *t*-test, with results considered significant at ** *p* < 0.01. Data represent the mean ± S.D., *n* = 3. (**D**) Senescent fibroblasts were administered with DMSO (0.01%) or sauchinone (1 µM) for 12 days. Use of DHR123 to detect mitochondrial ROS levels. Statistical analysis was performed using Student’s *t*-test, with results considered significant at ** *p* < 0.01. Data represent the mean ± S.D., *n* = 3. (**E**) Senescent fibroblasts were administered with DMSO (0.01%) or sauchinone (1 µM) for 12 days. Measurement of cell viability after 0, 4, 8, and 12 days of treatment. n.s. (not significant), two-way ANOVA followed by Bonferroni’s post-hoc test. Data represent the mean ± S.D., *n* = 3.

Previous studies have shown that pinusolide (a labdan diterpenoid), sauchinone (a lignan), and puerarin (an isoflavonoid) have anti-inflammatory and some antioxidant activities (Table 3). However, it is unknown how each compound affects mitochondrial ROS levels, so we evaluated the mitochondrial ROS levels after treating senescent fibroblasts with each compound. To determine the concentrations at which the three compounds were administered in senescent fibroblasts, we first conducted a literature search. Pinusolide derivatives used in the concentration range of 0–50 µM inhibited inducible nitric oxide synthase, which is involved in inflammation, starting from 5 µM [28]. In addition, sauchinone used in the concentration range of 0–40 µM induced the expression of heme oxidase-1, which inhibits inflammatory mediators starting at 3 µM [37]. Finally, puerarin used in the concentration range of 0–40 µM decreased the expression level of the inflammatory mediator starting at 1 µM [38]. Since the anti-inflammatory effects of pinusolide, sauchinone, or puerarin started at the concentration range of 1–5 µM, a concentration of 4 µM was selected for each compound. Each compound was administered to senescent fibroblasts at a concentration of 4 µM for 12 days. Then, the effect of each compound on the ROS levels was investigated using DHR123, which can specifically detect ROS in mitochondria [39]. Young fibroblasts served as a positive control. As expected, young fibroblasts had significantly lower mitochondrial ROS levels than senescent fibroblasts administered with DMSO [40] (Figure 1A). Pinusolide, sauchinone, and puerarin all significantly reduced mitochondrial ROS levels (Figure 1A). Sauchinone was selected as a candidate for further study, as it reduced mitochondrial ROS levels the most among the three compounds (Figure 1A).

The finding that sauchinone is effective in reducing ROS led us to determine the optimal concentration at which sauchinone reduces senescence-associated phenotypes. Since cell cycle arrest is one of the key features of senescence [42], we investigated the anti-aging effects of sauchinone based on whether it induces proliferation. To find the optimal concentration to induce proliferation, senescent fibroblasts were administered with sauchinone at concentrations of 0.25, 0.5, 1, 2, and 4 μM for 12 days. Young fibroblasts served as a positive control. As previously reported [1], young fibroblasts showed significantly increased proliferation compared to senescent fibroblasts administered with DMSO (Figure 1B). Furthermore, the senescent fibroblasts administered with various concentrations of sauchinone showed significantly increased proliferation compared to the senescent fibroblasts administered with DMSO (Figure 1B). Since the greatest effect on proliferation was observed at a concentration of 1 μM among the various concentrations of sauchinone. 1 μM sauchinone was selected as the optimal concentration to restore senescence-associated phenotypes.

Since we chose 1 μM sauchinone based on the cell proliferation data at one time point (day 12), we thought that measuring the cell proliferation beyond day 12 would support our results. To further support this, the cell proliferation of senescent fibroblasts administered with DMSO (0.01%) or sauchinone (1 μM) was measured at day 16. Compared to the DMSO control, senescent fibroblasts administered with 1 μM sauchinone showed significantly increased cell proliferation, even at day 16, further supporting our choice of 1 μM sauchinone as the optimal concentration (Figure 1C).

We then investigated whether the ROS-reducing effect was maintained at 1 μM sauchinone, since the selected concentration (1 μM) was different from the concentration used in the ROS-based screening (4 μM). Compared to the DMSO control, treatment with 1 μM sauchinone significantly reduced the mitochondrial ROS levels, suggesting that the ability of sauchinone to reduce mitochondrial ROS was consistent even at 1 μM (Figure 1D).

Next, we examined the toxicity of sauchinone at the selected concentration. Cell proliferation evaluates the rate at which cells divide in response to a drug [43], while cell viability evaluates the percentage of cells that are alive in response to a drug [44]. Therefore, to assess whether sauchinone is toxic to cells, cell viability was examined. Senescent fibroblasts administered with 1 μM sauchinone showed a similar viability to the senescent fibroblasts administered with DMSO, indicating that 1 μM sauchinone is not toxic to the cells (Figure 1E).

### 3.2. Restoration of Mitochondrial Function by Sauchinone in Senescent Fibroblasts

**Figure 2:**
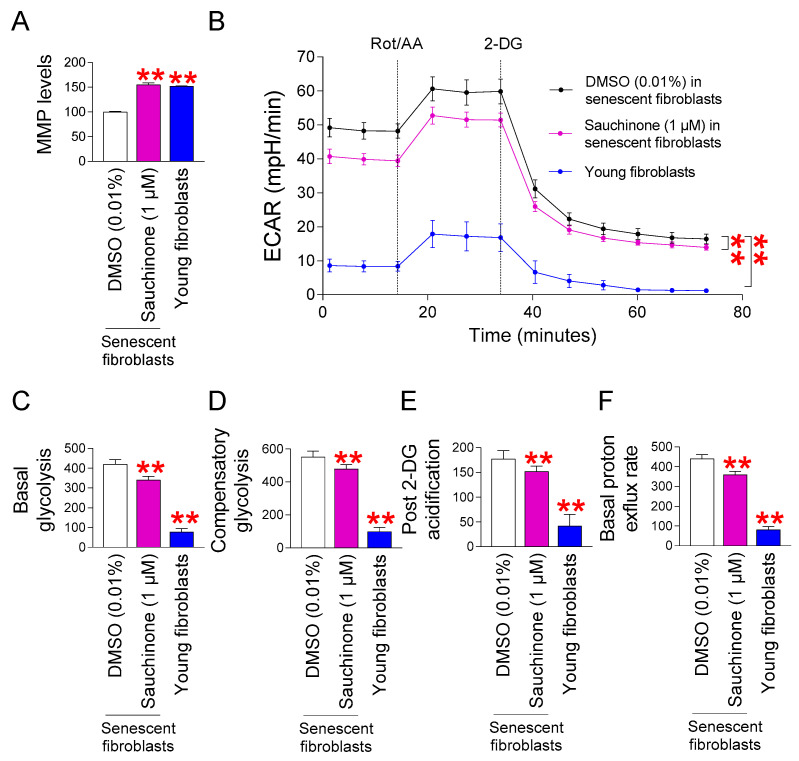
Restoration of mitochondrial function by sauchinone in senescent fibroblasts. (**A**) Senescent fibroblasts were administered with DMSO (0.01%) or sauchinone (1 µM) for 12 days. Use of JC–10 dye to measure mitochondrial membrane potential (MMP). Statistical analysis was performed using Student’s *t*-test, with results considered significant at ** *p* < 0.01. Data represent the mean ± S.D., *n* = 3. (**B**) Measurement of extracellular acidification rate (ECAR; mpH/min) after 12 days of treatment with DMSO (0.01%) or sauchinone (1 µM) (black line: DMSO–administered senescent fibroblasts, purple line: sauchinone-treated senescent fibroblasts, blue line: young fibroblasts). Statistical analysis was performed using two-way ANOVA followed by Bonferroni’s post hoc test, with results considered significant at ** *p* < 0.01. Data represent the mean ± S.D., *n* = 3. (**C**–**F**) Basal glycolysis (**C**), compensatory glycolysis (**D**), post–2–DG acidification (**E**), or basal proton exflux rate (**F**) was measured after 12 days of treatment with DMSO (0.01%) or sauchinone (1 µM). Young fibroblasts served as a positive control. Statistical analysis was performed using Student’s *t*-test, with results considered significant at ** *p* < 0.01. Data represent the mean ± S.D., *n* = 3.

Inefficiency of electron transport in ETC is a crucial factor in the ROS production in mitochondria [45]. This inefficiency causes ETC components to generate ROS as byproducts [45]. One of the mitochondrial damages caused by ROS is a decrease in MMP, which occurs due to the movement of protons from the mitochondrial matrix to the intermembrane space [46]. Inefficient electron transfer increases ROS production and decreases MMP, whereas efficient electron transfer decreases ROS production and increases MMP [47]. Since we observed that sauchinones decrease mitochondrial ROS levels, we investigated their effects on MMP. As previously reported [35], young fibroblasts showed a significantly increased MMP compared to senescent fibroblasts administered with DMSO (Figure 2A). Moreover, senescent fibroblasts administered with sauchinone showed significantly increased MMP compared to senescent fibroblasts administered with DMSO (Figure 2A).

MMP, a proton motive force, stimulates ATP synthesis through oxidative phosphorylation (OXPHOS) [48]. Young fibroblasts enable efficient ATP production via OXPHOS, reducing their dependence on glycolysis for energy, whereas senescent fibroblasts have impaired mitochondrial function, making them less able to generate ATP via OXPHOS, increasing their dependence on glycolysis for energy [49]. Since we discovered that sauchinone raised MMP, we investigated how auquinone affected the senescent fibroblasts’ reliance on glycolysis. The extracellular acidification rate (ECAR) following the sequential infusion of 2-deoxy-D-glucose (2-DG), and rotenone/antimycin A (Rot/AA) was used to determine the glycolysis rate. In particular, the basal glycolysis rate (before to Rot/AA addition), the compensatory glycolysis rate (post-Rot/AA addition), and the post-2-DG acidification (post-2-DG addition) were evaluated using serially collected ECAR measurements. As previously reported [35], young fibroblasts showed a significantly lower ECAR compared to the senescent fibroblasts administered with DMSO (Figure 2B). Moreover, senescent fibroblasts administered with sauchinone showed a significantly lower ECAR compared to the senescent fibroblasts administered with DMSO, suggesting that sauchinone decreased the overall glycolysis rate (Figure 2B).

Specifically, basal glycolysis levels were significantly reduced in young fibroblasts and sauchinone-treated senescent fibroblasts compared to DMSO-treated senescent fibroblasts, suggesting that the rate of converting glucose to lactate was reduced by sauchinone [50] (Figure 2C).

Compensatory glycolysis in young fibroblasts and sauchinone-treated senescent fibroblasts was significantly decreased by the addition of a mitochondrial inhibitor (Rot/AA), which inhibits OXPHOS and induces a compensatory shift toward glycolysis, suggesting that sauchinone reduced the maximal glycolytic capacity [50] (Figure 2D).

Post-2-DG acidification in young fibroblasts and sauchinone-treated senescent fibroblasts was significantly reduced when 2-DG was added, which inhibits glycolysis (Figure 2E). These data suggest that sauchinone reduces the residual glycolysis that is not fully inhibited by 2-DG [50].

After glycolysis, protons not required for cellular respiration are used to produce lactic acid [51]. Lactic acid fermentation is an inefficient energy metabolic process that produces a small amount of energy [51]. When pyruvate is converted to lactic acid, more protons are produced [52]. Thus, the basal proton flux rate was assessed to determine how sauchinone affects the glycolysis rate. The basal proton flux rate of young fibroblasts and sauchinone-treated senescent fibroblasts was significantly reduced compared to DMSO-treated senescent fibroblasts (Figure 2F). These results demonstrate that sauchinone reduces the rate of glycolysis, suggesting a restoration of mitochondrial function.

### 3.3. Sauchinone Removes Dysfunctional Mitochondria Through Mitophagy

**Figure 3:**
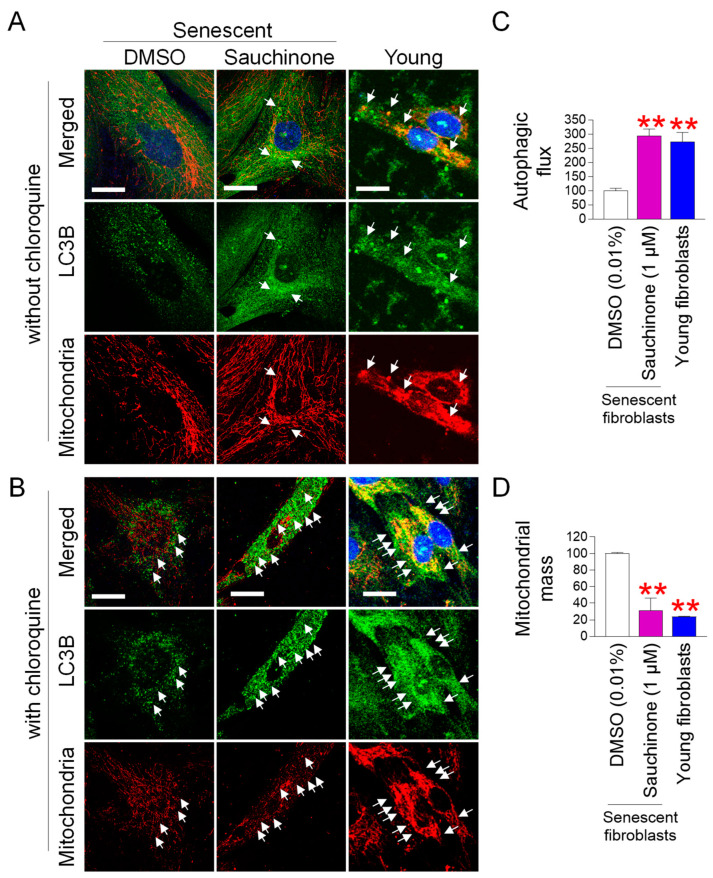
Sauchinone removes dysfunctional mitochondria through mitophagy. (**A**,**B**) Senescent fibroblasts were administered with DMSO (0.01%) or sauchinone (1 µM) for 12 days. Young fibroblasts served as a positive control. Immunostaining for LC3B (green) and mitochondria (red) before chloroquine (**A**) or after chloroquine treatment (**B**). Scale bar 10 μm. White arrow indicates mitophagy. (**C**,**D**) Measurement of autophagic flux (**C**) and mitochondrial mass (**D**) after 12 days of treatment with DMSO (0.01%) or sauchinone (1 µM). Young fibroblasts served as a positive control. Statistical analysis was performed using Student’s *t*-test, with results considered significant at ** *p* < 0.01. Data represent the mean ± S.D., *n* = 3.

The discovery that sauchinone restores mitochondrial function led us to investigate how sauchinone removes dysfunctional mitochondria in senescent fibroblasts. We hypothesized that sauchinone removes dysfunctional mitochondria by triggering mitophagy, which removes damaged mitochondria while maintaining normal mitochondrial function [53]. Therefore, we investigated how sauchinone affects mitophagy. Since autophagosomes remove defective mitochondria [54], we investigated mitophagy using the colocalization of the autophagosomal membrane protein microtubule-associated protein 1A/1B-light chain 3B (LC3B) with mitochondria [55]. Colocalization between LC3B and mitochondria was observed in young fibroblasts but was barely observed in DMSO-treated senescent fibroblasts (Figure 3A; white arrows). However, following sauchinone treatment in senescent fibroblasts, colocalization reappeared (Figure 3A; white arrows). To confirm the role of sauchinone in mitophagy, we co-treated cells with chloroquine (CQ), which limits autophagic flux by disrupting lysosomal pH [56]. As expected, CQ treatment increased autophagosome accumulation (LC3B, green) and the colocalization of LC3B with mitochondria in young fibroblasts and sauchinone-treated senescent fibroblasts but not in DMSO-treated senescent fibroblasts (Figure. 3B; white arrows). The increase in colocalization was accompanied by an increase in autophagic flux and a decrease in mitochondrial mass in sauchinone-treated senescent fibroblasts, similar to young fibroblasts, suggesting that mitophagy activation was mediated by sauchinone (Figure 3C,D).

### 3.4. Sauchinone Rejuvenates Senescence-Associated Phenotypes

**Figure 4:**
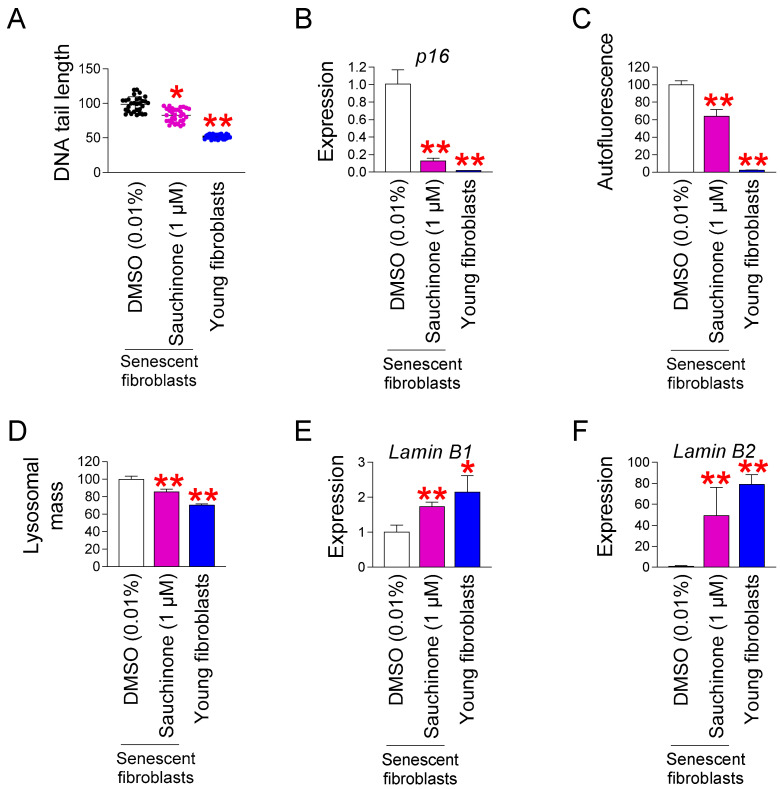
Sauchinone rejuvenates senescence-associated phenotypes. (**A**) Measurement of DNA tail length after 12 days of treatment with DMSO (0.01%) or sauchinone (1 µM). Young fibroblasts served as a positive control. Each dot represents the length of a DNA tail. Statistical analysis was performed using Student’s *t*-test, with results considered significant at * *p* < 0.05 and ** *p* < 0.01. Data represent the mean ± S.D., *n* = 50. (**B**) Expression level of *p16* after 12 days of treatment with DMSO (0.01%) or sauchinone (1 µM). Young fibroblasts served as a positive control. Statistical analysis was performed using Student’s *t*-test, with results considered significant at ** *p* < 0.01. Data represent the mean ± S.D., *n* = 3. (**C**,**D**) Measurement of autofluorescence (**C**) and lysosomal mass (**D**) after 12 days of treatment with DMSO (0.01%) or sauchinone (1 µM). Young fibroblasts served as a positive control. Statistical analysis was performed using Student’s *t*-test, with results considered significant at ** *p* < 0.01. Data represent the mean ± S.D., *n* = 3. (**E**,**F**) Expression level of *lamin B1* (**E**) and *lamin* B2 (**F**) after 12 days of treatment with DMSO (0.01%) or sauchinone (1 µM). Young fibroblasts served as a positive control. Statistical analysis was performed using Student’s *t*-test, with results considered significant at * *p* < 0.05 and ** *p* < 0.01. Data represent the mean ± S.D., *n* = 3.

Mitochondrial functional recovery serves as a prerequisite for rejuvenating senescence [34,49,57,58,59]. The discovery that sauchinone restores mitochondrial function led us to examine how it might affect senescence-associated phenotypes. ROS can damage DNA directly or indirectly by damaging the proteins necessary for DNA stability [60]. Because we observed a decrease in ROS mediated by sauchinone, we evaluated the effect of sauchinone on DNA damage. DNA double-strand breaks (DSBs) were assayed to quantify the amount of DNA damage [35]. As previously reported [35], young fibroblasts showed significantly lower DNA DSBs compared to DMSO-treated senescent fibroblasts (Figure 4A). Moreover, senescent fibroblasts administered with sauchinone showed significantly lower DNA DSBs compared to DMSO-treated senescent fibroblasts (Figure 4A).

Two important pathways that regulate cell cycle arrest are p16/retinoblastoma (RB) and p53/p21 which are a characteristic of senescence [61]. While the p53/p21 is important in the early stage of senescence, the p16/RB plays an essential role in maintaining senescence [62]. Among the two pathways, we chose the p16/RB and specifically examined the expression of *p16*, an upstream pathway of RB. The expression of *p16* in young fibroblasts was significantly lower than that in DMSO-treated senescent fibroblasts, as previously reported [35] (Figure 4D). The treatment of senescent fibroblasts with sauchinone significantly reduced the expression of *p16* compared to that in senescent fibroblasts administered with DMSO, indicating sauchinone-mediated cell cycle progression (Figure 4B).

Next, we examined the levels of intracellular lipofuscin, the main characteristics associated with senescence [63]. Lipofuscin levels were examined by measuring the amount of autofluorescence [63]. Young fibroblasts showed significantly less autofluorescence than DMSO-treated-senescent fibroblasts, as previously reported [35] (Figure 4C). In addition, sauchinone-treated senescent fibroblasts showed significantly less autofluorescence than DMSO-treated senescent fibroblasts (Figure 4C).

One kind of autofluorescent substance that progressively builds up in lysosomes is lipofuscin. Lipofuscin-filled lysosomes decrease lysosomal activity by acting as a sink for freshly produced hydrolytic enzymes [64]. In order to make up for the activity, lysosomal mass increases in response to the decreased activity [60]. To ascertain if sauchinone influences lysosomal activity, we examined the lysosomal mass. Young fibroblasts showed significantly less lysosomal mass than DMSO-treated senescent fibroblasts, as previously reported [35] (Figure 4D). In addition, sauchinone-treated senescent fibroblasts showed significantly less lysosomal mass than DMSO-treated senescent fibroblasts (Figure 4D).

Lamin B1 and lamin B2 are two B-type lamin proteins, which are components of the nuclear envelope. Lamin B1 expression is lost when senescence is induced by replicative exhaustion or oncogene expression, so lamin B1 has been used as a novel marker for identifying senescent cells [65,66]. Furthermore, lamin B2 expression is decreased during the aging process of human skin [67]. Therefore, we investigated the changes in lamin B1 and B2 expression to determine the role of sauchinone in improving senescence. Young fibroblasts showed significantly higher *lamin B1/B2* expression than DMSO-treated senescent fibroblasts (Figure 4E,F). Sauchinone-treated senescent fibroblasts showed significantly higher *lamin B1/B2* expression than DMSO-treated senescent fibroblasts, suggesting that sauchinone restored *lamin B1/B2* expression (Figure 4E,F).

### 3.5. Identification of VAMP8 as a Key Regulator in Sauchinone-Induced ROS Reduction

**Figure 5:**
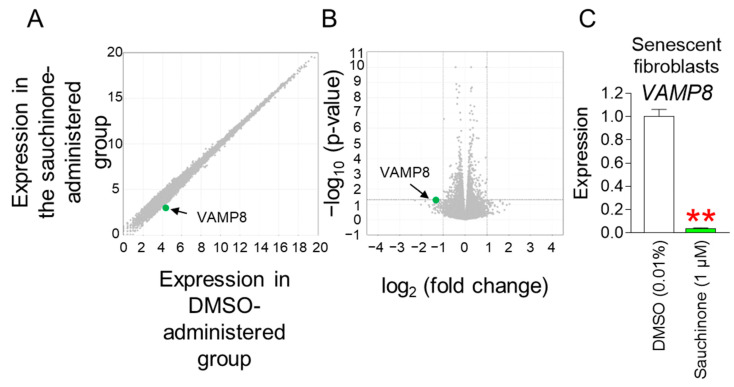
Identification of *VAMP8* as a key regulator in sauchinone-induced ROS reduction. (**A**) Graph showing expression in sauchinone-administered senescent fibroblasts versus expression in DMSO-administered senescent fibroblasts. *VAMP8*, which was selected as a key candidate (blue dot). Gray dot: non-selected gene. (**B**) Volcano plot showing log_2_ (fold change) versus −log_10_ (*p*-value). Green dot: *VAMP8*. Gray dot: non-selected gene. (**C**) After 12 days of treatment with DMSO (0.01%) or sauchinone (1 µM) in senescent fibroblasts, expression levels of *VAMP8* were evaluated. Statistical analysis was performed using Student’s *t*-test, with results considered significant at ** *p* < 0.01. Data represent the mean ± S.D., *n* = 3.

The discovery that sauchinone has anti-aging effects led us to investigate the mechanism by which sauchinone reverses senescence. To investigate the underlying mechanism, RNA sequencing was performed after administering senescent fibroblasts with DMSO or sauchinone for 12 days. Differentially expressed genes (DEGs) were derived using transcriptome-sequencing data. Through DEG analysis, 55 genes that were altered more than two-fold compared to the DMSO control were identified (Supplementary Table S1). A candidate approach was used to identify the genes involved in ROS production or inhibition among 55 genes. Among the 55 genes, *vesicle-associated membrane protein 8* (*VAMP8*; Accession Number: NM_003761) was known to have a decreasing effect on ROS generation when its expression was decreased [68]. Therefore, we hypothesized that sauchinone reduces ROS levels by decreasing *VAMP8* expression. To confirm this hypothesis, we first examined changes in *VAMP8* expression using RNA-sequencing results. According to the DEG analysis results, *VAMP8* expression was reduced 2.59-fold when administered with sauchinone compared to the DMSO control group (green dot, Figure 5A and Supplementary Table S1). The fold change of *VAMP8* was statistically significant, as shown in the volcano plot showing the fold change and *p*-value (green dot, Figure 5B and Supplementary Table S1). To further support the hypothesis, the DEG analysis results were confirmed by qPCR. The qPCR also showed that *VAMP8* expression was significantly reduced in the sauchinone treatment group compared to DMSO (Figure 5C). As a result, *VAMP8* was identified as a key candidate for sauchinone-mediated anti-senescence.

### 3.6. VAMP8 Knockdown Reduces Mitochondrial ROS Levels and Restores Mitochondrial Function

**Figure 6:**
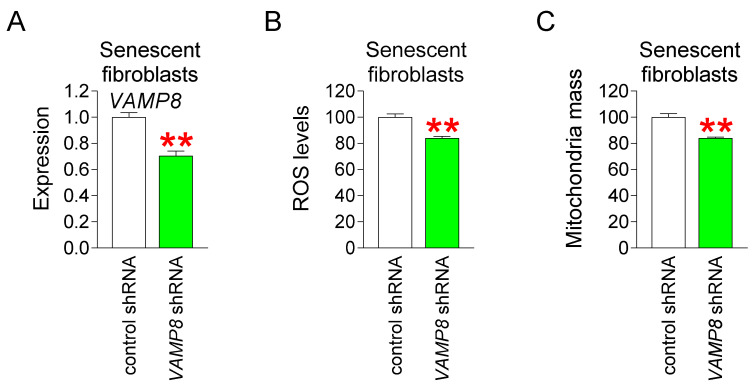
*VAMP8* knockdown reduces mitochondrial ROS levels and restores mitochondrial function. (**A**) Compared with the control group, *VAMP8* shRNA group showed a significant decrease in *VAMP8* expression. Statistical analysis was performed using Student’s *t*-test, with results considered significant at ** *p* < 0.01. Data represent the mean ± S.D., *n* = 3. (**B**) Compared with the control group, *VAMP8* shRNA group showed a significant decrease in mitochondrial ROS levels. Statistical analysis was performed using Student’s *t*-test, with results considered significant at ** *p* < 0.01. Data represent the mean ± S.D., *n* = 3. (**C**) Compared with the control group, *VAMP8* shRNA group showed a significant decrease in mitochondrial mass. Statistical analysis was performed using Student’s *t*-test, with results considered significant at ** *p* < 0.01. Data represent the mean ± S.D., *n* = 3.

Our finding that *VAMP8* is a candidate gene for sauchinone-induced senescence improvement led us to examine whether downregulating *VAMP8* could have the same anti-senescence effect as sauchinone. After making a lentiviral system for knocking down *VAMP8*, senescent fibroblasts were infected with a lentivirus expressing control shRNA or expressing shRNA targeting *VAMP8*. The *VAMP8* shRNA group showed effective shRNA-induced *VAMP8* knockdown, as shown by a significant decrease in *VAMP8* expression when compared with the control shRNA group (Figure 6A).

We then examined how *VAMP8* knockdown affected mitochondrial ROS levels. As shown by the significant decrease in mitochondrial ROS levels in the *VAMP8* shRNA group compared to the control shRNA, *VAMP8* knockdown showed the same ROS-reducing effect as sauchinone (Figure 6B).

Since ROS-induced mitochondrial deterioration causes the mitochondrial mass to increase as a compensatory strategy [69,70], we examined the effect of *VAMP8* knockdown on mitochondrial mass. The *VAMP8* shRNA group showed a significant decrease in mitochondrial mass compared to the control shRNA group (Figure 6C). This suggests that the effect of *VAMP8* knockdown on mitochondrial mass reduction is similar to that of sauchinone.

## 4. Discussion

The mitochondria are organelles that use more than 90% of the oxygen within cells and generate ROS as byproducts during cellular respiration [71]. Complexes of the mitochondrial ETC convert 1–5% of the oxygen consumed by mitochondria into ROS [45]. Mitochondrial dysfunction due to senescence results in decreased activities in the complexes of the mitochondrial ETC [72,73]. Specifically, a lower activity of complex I impedes efficient electron transport and leaks electrons to oxygen, which leads to more rapid ROS production [74]. Increased ROS production in mitochondria exacerbates ETC damage, starting a vicious cycle in which mitochondrial ROS production increases [75]. This vicious cycle leads to a decline in cellular organelle function and ultimately senescence [76]. This causal relationship emphasizes that reducing mitochondrial ROS production is an important strategy for reversing senescence [75]. Here, we identify a novel mechanism by which sauchinone restores mitochondrial function and thereby reduces mitochondrial ROS production. The increase in MMP by sauchinone suggests increased proton transport, suggesting efficient electron transport within the ETC [77]. Efficient electron transport by sauchinone reduces electron leak in the ETC, thereby reducing ROS production [78]. Furthermore, we find that sauchinone reduces the dependence on glycolysis, indirectly suggesting that sauchinone restores mitochondrial function. The mitochondrial functional recovery by sauchinone was accompanied by a reversal of senescence-related phenotypes. Here, we find that sauchinone rejuvenates senescence by reducing ROS production in the mitochondria. We propose that this novel mechanism may be a fundamental step toward the development of therapeutics for aging.

Autophagy maintains cellular homeostasis by removing damaged organelles and proteins [79]. However, as senescence progresses, the efficiency of autophagy decreases, leading to the accumulation of cellular waste and further accelerated senescence [80]. In this study, we found that sauchinone restores autophagy activity, as evidenced by the increase in sauchinone-mediated autophagic flux. The restoration of autophagy by sauchinone is further supported by the finding that sauchinone promotes mitophagy to specifically remove damaged mitochondria. The activation of mitophagy led to a decrease in dysfunctional mitochondria, which in turn, decreased mitochondrial ROS levels. As far as we are aware, these results are the first to elucidate how sauchinone removes dysfunctional mitochondria in senescent fibroblasts. Here, we propose a novel mechanism by which sauchinone removes dysfunctional mitochondria by activating the autophagy system, thereby reducing mitochondrial ROS and rejuvenating senescence.

Secondary metabolites found in plants act as antioxidants due to their structural characteristics [81]. Representative substances include quercetin, resveratrol, and curcumin [82]. Quercetin has multiple hydroxyl groups that can neutralize ROS by donating hydrogen atoms, making it an effective antioxidant [83]. In addition, resveratrol, a stilbene, has hydroxyl groups that contribute to radical-scavenging ability and is known to inhibit lipid peroxidation [84,85]. Curcumin, a flavonoid, is a polyphenol found in turmeric that has a unique diketone structure and multiple phenolic hydroxyl groups, which can effectively neutralize free radicals and suppress oxidative stress [84,85]. Pinusolide, sauchinone, and puerarin, which were used in this study, have structural characteristics. Pinusolide has a stilbene skeleton, which has a high electron transfer ability, and it can easily donate electrons because it has two hydroxyl groups [86]. Sauchinone has a lignan skeleton composed of four hydroxyl groups and two ether bonds [87]. Puerarin can stabilize radicals through the isoflavone resonance structure and one ether bond [88]. Here, we examined the ability of pinusolide, sauchinone, and puerarin to reduce mitochondrial ROS levels. All three secondary metabolites were effective in reducing mitochondrial ROS levels, but sauchinone showed the highest antioxidant capacity. This is because sauchinone has distinct structural characteristics compared to pinusolide and puerarin. Comparing sauchinone and pinusolide, both have a hydroxyl group that reacts directly with ROS via electrons from the hydroxyl group [89]. However, sauchinone has four hydroxyl groups, while pinusolide has two. Therefore, the four hydroxyl groups in sauchinone may eliminate more free radicals than pinusolide. Comparing sauchinone and puerarin, both have an ether with a lone electron pair on the oxygen, which helps stabilize free radicals or ROS [90]. However, sauchinone has two ether groups, while puerarin has one. Therefore, the two ether bonds in sauchinone may increase the overall electron density, allowing it to have a higher antioxidant capacity than puerarin. In summary, our results suggest that the antioxidant activity of the secondary metabolites varies depending on the number of ether and hydroxyl groups. We propose that further modification of sauchinone may further increase the antioxidant capacity. However, we acknowledge that further experiments are needed to confirm this hypothesis.

The antioxidant and senescence-rejuvenating effect of sauchinone may be due to multiple mechanisms working together. For example, Apigenin, a flavonoid found in many fruits and vegetables, contains multiple hydroxyl groups and double bonds in its C-ring, which can effectively scavenge ROS [91]. It also regulated gene expression, specifically targeting the genes involved in oxidative stress and inflammation, such as *nuclear factor erythroid 2-related factor 2*, which is important for antioxidant defense mechanisms [92]. Extending the relevance of these findings, baicalin, a flavone glycoside found in *Scutellaria baicalensis*, has potent antioxidant effects due to its phenolic hydroxyl group, which can donate electrons and neutralize ROS [93]. Baicalin also downregulates the expression of inflammatory genes, such as *tumor necrosis factor-alpha* and *interleukin-6*, which helps reduce overall oxidative damage [94,95]. Here, we found that sauchinone appears to combine the structural properties that can scavenge free radicals with the ability to regulate the expression of *VAMP8*. This combination of mechanisms may explain why sauchinone exhibits stronger antioxidant effects than other natural extracts, such as pinusolide or puerarin. However, to fully understand these mechanisms, future studies should focus on exploring the mechanisms by which *VAMP 8* controls mitochondrial ROS levels.

Current research on aging focuses on senotherapy, which treats aging like any other disease [96]. Senotherapy can be classified into two main types: senolytics and senomorphics. Senolytics selectively kill senescent cells that can impair the function of normal cells [97], whereas senomorphics improve the phenotype of senescent cells toward that of younger cells by modulating various signaling pathways [98]. Senolytics, which selectively eliminate senescent cells, are known to be effective in reversing senescence but may have potential side effects that impair the function of normal cells [99]. For example, quercetin, a widely used senolytic drug, significantly reduced senescence-related β-gal activity and inflammatory cytokines by selectively killing senescent cells [100]. However, quercetin affected the expression levels of polymerase gamma and mitochondrial transcription factor A genes, consequently resulting in suppression of the mtDNA copy number [101]. This suppression resulted in lower expression levels of the gene encoding the mitochondrial-specific polymerase, thereby inhibiting mitochondrial anabolism [101]. These potential side effects highlight the need for a careful application of senolytics as senotherapies [99]. Senomorphs have emerged as an alternative therapeutic strategy to minimize these side effects [98]. Metformin, which has ROS-scavenging activity, is one of the most widely used senomorphics [102]. Metformin restored senescence-related phenotypes by reducing oxidative stress in senescent adipose stromal cells [103]. Metformin-induced senescence rejuvenation improved adipogenic capacity and insulin sensitivity [103]. The importance of senomorphics for senescence rejuvenation is further supported by studies showing that metformin extends lifespan in *C. elegans* and mice [104]. In this study, we found that sauchinone induces senescence rejuvenation, not by inducing selective death of senescent fibroblasts but rather by increasing the proliferation of senescent fibroblasts. Sauchinone-mediated senescence rejuvenation was further emphasized by the restoration of mitochondrial function and autophagy activity to levels that are similar to young fibroblasts. Consequently, the restoration of function to the level of young fibroblasts led to the restoration of various senescence-associated phenotypes. Based on these results, we conclude that sauchinone acts as senomorphics rather than senolytics.

## 5. Conclusions

In summary, we found that sauchinone was effective in reducing mitochondrial ROS levels in senescent fibroblasts. The effect of sauchinone on ROS reduction led to the restoration of mitochondrial function and senescence-associated phenotypes. Moreover, the senescence-rejuvenating effect of sauchinone was achieved through the downregulation of *VAMP8*. *VAMP8* knockdown also showed similar effects to sauchinone. Our results provide a novel mechanism by which sauchinone rejuvenates senescence by reducing mitochondrial ROS levels. Furthermore, our results revealed that the downregulation of *VAMP8* by sauchinone is an important factor in improving senescence. Future studies evaluating the efficacy of sauchinone in clinical settings will contribute to the development of novel therapeutics for aging and aging-related diseases.

## Figures and Tables

**Table 1:** List of primers.

Target	Orientation	Sequence (5′–3′)	Size (bp)
*36B4* (Accession number: NM_053275)	Forward	CAGCAAGTGGGAAGGTGTAATCC	23
	Reverse	CCCATTCTATCATCAACGGGTACAA	25
*p16* (Accession number: NM_000077.5)	Forward	CTCGTGCTGATGCTACTGAGGA	22
	Reverse	GGTCGGCGCAGTTGGGCTCC	20
*Lamin A* (Accession number: NM_170707)	Forward	ATGAGGACCAGGTGGAGCAGTA	22
	Reverse	ACCAGGTTGCTGTTCCTCTCAG	22
*Lamin B1* (Accession number: NM_005573)	Forward	GAGAGCAACATGATGCCCAAGTG	23
	Reverse	GTTCTTCCCTGGCACTGTTGAC	22
*Lamin B2* (Accession number: NM_032737)	Forward	AGAAGTCCTCGGTGATGCGTGA	22
	Reverse	CATCACGTAGCAGCCTCTTGAG	22
*VAMP8* (Accession number: NM_003761)	Forward	AAGGTGGAGGAAATGATCTGGTG	23
	Reverse	GGAGGGAGTTAAGAATATTATGACCCAGAAT	31

**Table 2:** Details of primers used in making *VAMP8* shRNA.

Target	Orientation	Sequence (5′–3′)	Size (bp)
*VAMP8* shRNA (1)	Forward	CCGGGTGGAGGGAGTTAAGAATATTTTCAAGAGAAATATTCTTAACTCCCTCCACTTTTTG	61
	Reverse	AATTCAAAAAGTGGAGGGAGTTAAGAATATTTTCAAGAGAAATATTCTTAACTCCCTCCAC	61
*VAMP8* shRNA (2)	Forward	CCGGGCCACTGGTGCCTTCTCTTAATTCAAGAGATTAAGAGAAGGCACCAGTGGCTTTTTG	61
	Reverse	AATTCAAAAAGCCACTGGTGCCTTCTCTTAATTCAAGAGATTAAGAGAAGGCACCAGTGGC	61
*VAMP8* shRNA (3)	Forward	CCGGGCCAGTGAAGGTGGAGGAAATTTCAAGAGAATTTCCTCCACCTTCACTGGCTTTTTG	61
	Reverse	AATTCAAAAAGCCAGTGAAGGTGGAGGAAATTTCAAGAGAATTTCCTCCACCTTCACTGGC	61

**Table 3:** Compounds library used for screening in this study.

Compound Name	Structure	Molecular Formula	Bioactivity
		Molecular Weight (Da)	
Pinusolide	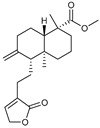	C_21_H_30_O_4_	Anti-inflammation [28]
		346.46	
Sauchinone	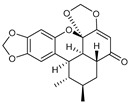	C_20_H_20_O_6_	Anti-inflammation and antioxidants [29]
		356.37	
Puerarin	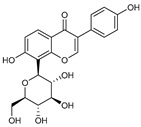	C_21_H_20_O_9_	Anti-inflammation and antioxidants [41]
		416.38	

## Data Availability

The original contributions presented in the study are included in the article, further inquiries can be directed to the corresponding authors.

## Supplementary Materials

The following supporting information can be downloaded at https://www.mdpi.com/article/10.3390/antiox14030259/s1: Figure S1. ^1^H–NMR spectrum (600 MHz, CD_3_OD) of pinusolide isolated from *Biota orientalis*. Figure S2. ^13^C–NMR spectrum (150 MHz, CD_3_OD) of pinusolide isolated from *Biota orientalis.* Figure S3. ^1^H–NMR spectrum (600 MHz, CDCl_3_) of sauchinone isolated from *Saururus chinensis.* Figure S4. ^13^C–NMR spectrum (150 MHz, CDCl_3_) of sauchinone isolated from *Saururus chinensis.* Figure S5. ^1^H–NMR spectrum (600 MHz, CD_3_OD) of puerarin isolated from *Pueraria lobata.* Figure S6. ^13^C–NMR spectrum (150 MHz, CD_3_OD) of puerarin isolated from *Pueraria lobata.* Figure S7. ESI-MS spectrum of sauchinone. Figure S8. High-performance liquid chromatography chromatogram of sauchinone for purity verification. Table S1. List of 55 genes significantly altered more than two fold in the sauchinone group compared to the DMSO group.
