# Supplementary material for: Sauchinone Ameliorates Senescence Through Reducing Mitochondrial ROS Production
Source: Antioxidants (Basel). 2025 Feb 24;14(3):259. doi: 10.3390/antiox14030259 (PMC11939387; doi:10.3390/antiox14030259)
Supplement: Supplementary file 1 [file antioxidants-14-00259-s001.zip › antioxidants-3422907-supplementary.pdf]

Supplementary information

Table S1. List of 55 genes significantly altered more than two fold in the sauchinone group compared to the DMSO group.

Gene symbol	Description	Relative expression (Sauchinone/DMSO)	p-value (Sauchinone/DMSO)
ABCB5	ATP binding cassette subfamily B member 5	-2.219670	0.003443415
BHLHE22	basic helix-loop-helix family member e22	2.014191	0.039054281
BMS1P4	BMS1 pseudogene 4	-2.236900	0.048016288
CCDC62	coiled-coil domain containing 62	-2.409483	0.03625715
CH25H	cholesterol 25-hydroxylase	-2.103472	0.025957065
CNFN	cornifelin	2.338934	0.041038943
CSF2RA	colony stimulating factor 2 receptor subunit alpha	2.271414	0.009731348
DIRAS2	DIRAS family GTPase 2	2.763108	0.017785852
DIRAS3	DIRAS family GTPase 3	2.069324	0.008093383
FGF17	fibroblast growth factor 17	2.334923	0.034209014
GDF10	growth differentiation factor 10	2.028970	0.033778413
HMGA1P8	high mobility group AT-hook 1 pseudogene 8	-2.812468	0.025502347
IGFN1	immunoglobulin like and fibronectin type III domain containing 1	-2.269486	0.003877647
IRAIN	IGF1R antisense imprinted non-protein coding RNA	2.008232	0.037967221
ISY1-RAB43	ISY1-RAB43 readthrough	-2.760907	0.005223227
LINC00115	long intergenic non-protein coding RNA 115	-2.149685	0.004053179

LINC01118	long intergenic non-protein coding RNA 1118	2.598710	0.035565203
LINC01232	long intergenic non-protein coding RNA 1232	-2.647647	0.024218461
LOC100289230	uncharacterized LOC100289230	-2.352187	0.017298302
LOC100289361	uncharacterized LOC100289361	-2.153034	0.022238919
LOC100420528	CTD nuclear envelope phosphatase 1 pseudogene	2.052441	0.043195453
LOC100506472	uncharacterized LOC100506472	-2.281932	0.030125272
LOC101928529	uncharacterized LOC101928529, transcript variant X1	3.423598	0.031784915
LOC101928674	uncharacterized LOC101928674	2.277147	0.029319566
LOC101929703	uncharacterized LOC101929703	-3.401234	0.003832293
LOC105370854	uncharacterized LOC105370854, transcript variant X2	2.160624	0.030257473
LOC105373953	uncharacterized LOC105373953, transcript variant X3	-2.116746	0.046322759
LOC105378936	uncharacterized LOC105378936	-2.193556	0.017173417
LOC107984852	uncharacterized LOC107984852	-2.028718	0.04960371
LOC107984969	uncharacterized LOC107984969	-2.294802	0.039236674
MANEA-DT	MANEA divergent transcript	-2.374470	0.042270026

MCM3AP-AS1	MCM3AP antisense RNA 1	-2.028849	0.002397306
PCDHA11	protocadherin alpha 11	2.962089	3.32621E-06
PCDHGC4	protocadherin gamma subfamily C, 4	-2.310585	0.016360629
PLPPR3	phospholipid phosphatase related 3	2.967446	0.011673808
PTPRO	protein tyrosine phosphatase receptor type O	2.395884	0.009282542
RFPL4B	ret finger protein like 4B	2.442991	0.032453234
RHBDL3	rhomboid like 3	2.100314	0.012342167
RN7SL2	RNA component of signal recognition particle 7SL2	-2.117101	1.99781E-06
RN7SL3	RNA component of signal recognition particle 7SL3	-3.008765	0.022119684
RN7SL4P	RNA, 7SL, cytoplasmic 4, pseudogene	-2.098832	0.043483745
RUNDC3A	RUN domain containing 3A	2.107282	0.003683205
SNORD16	small nucleolar RNA, C/D box 16	-2.481630	0.047680096
SPAG17	sperm associated antigen 17	-2.033224	0.019621919
STAG3L3	stromal antigen 3-like 3 (pseudogene)	4.886532	3.76611E-06
TAGAP	T cell activation RhoGTPase activating protein	-2.406461	0.03028408
TDRKH-AS1	TDRKH antisense RNA 1	-2.366894	0.037172484
TENM2	teneurin transmembrane protein 2	2.285437	0.009038488
TMED2-DT	TMED2 divergent transcript	-2.091964	0.021111387

TRIM63	tripartite motif containing 63	-2.341206	0.008669315
TVP23C-CDRT4	TVP23C-CDRT4 readthrough	-22.497575	8.14661E-19
VAMP8	vesicle associated membrane protein 8	-2.588983	0.044331103
VSTM5	V-set and transmembrane domain containing 5	2.111285	0.035444409
WBP2NL	WBP2 N-terminal like	-2.689573	0.039447109
ZASP	ZO-2 associated speckle protein	4.602668	0.007842058

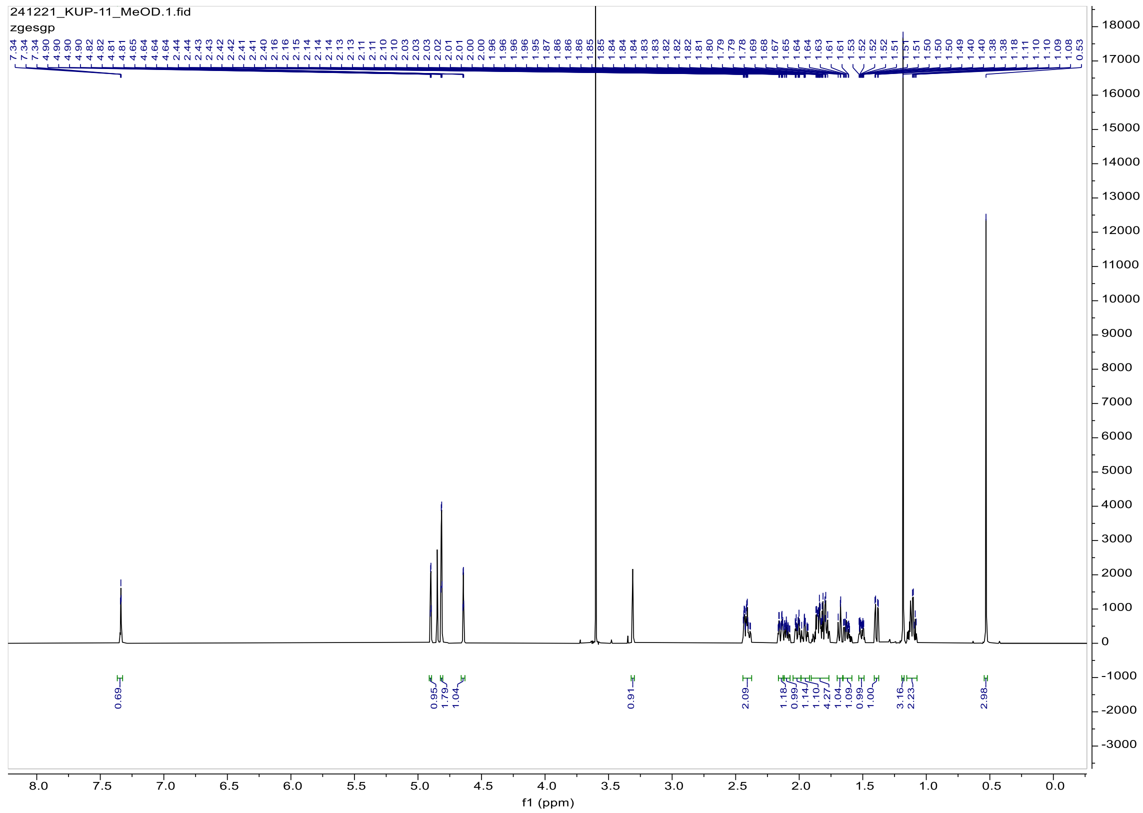

Figure S1. ^{1}H–NMR spectrum (600 MHz, CD_3OD) of pinusolide isolated from *Biota orientalis*.

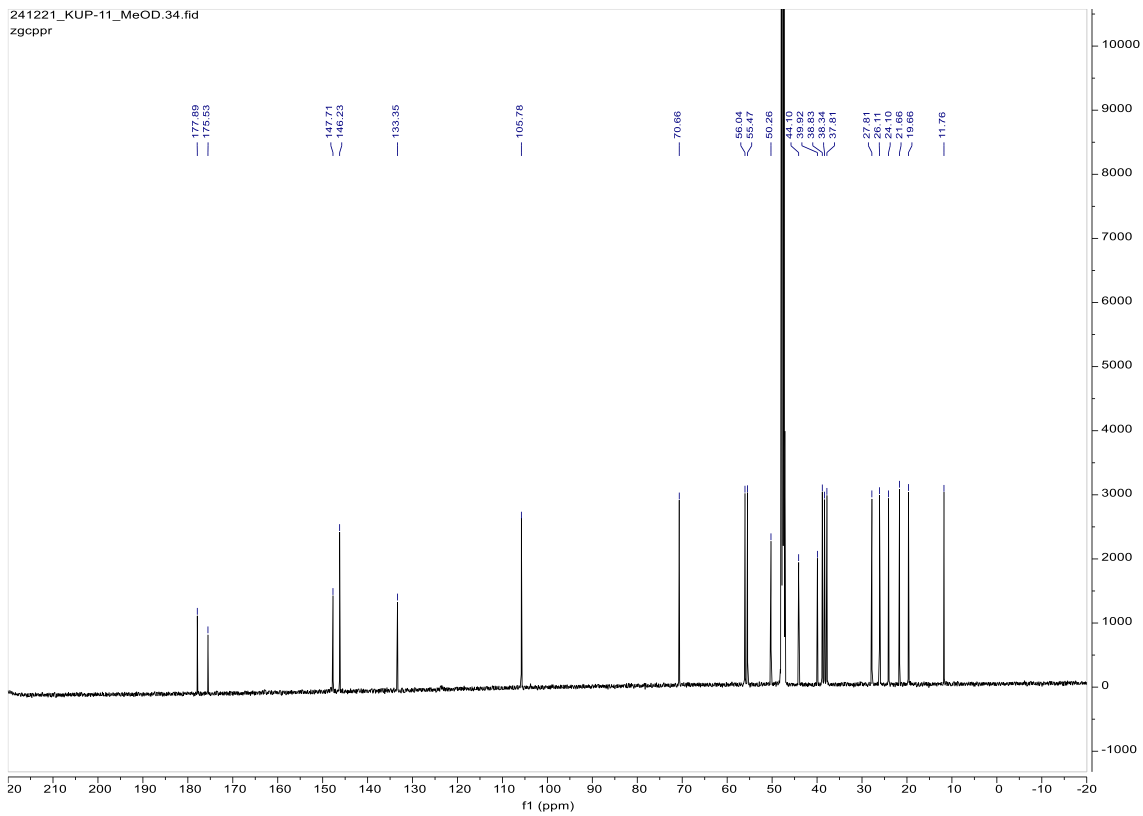

Figure S2. ^{13}C–NMR spectrum (150 MHz, CD_3OD) of pinusolide isolated from *Biota orientalis*.

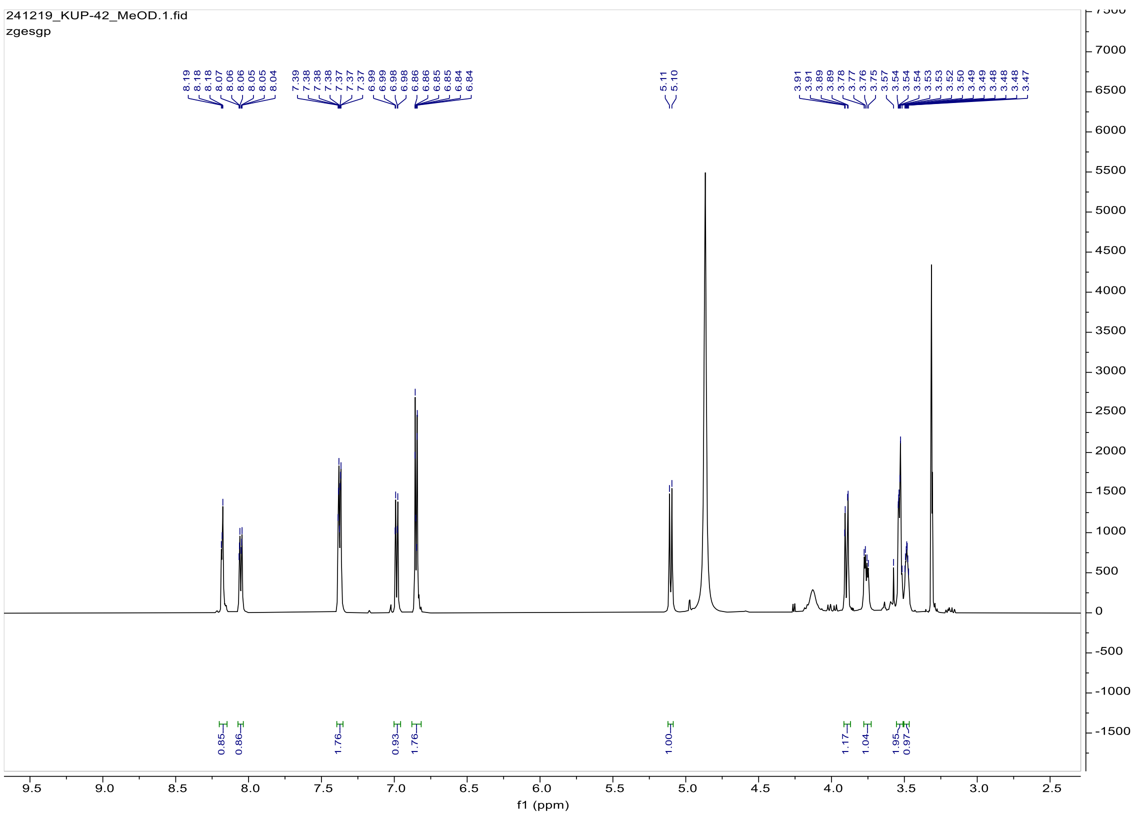

Figure S3. ^{1}H–NMR spectrum (600 MHz, CD_3OD) of puerarin isolated from *Pueraria lobata*.

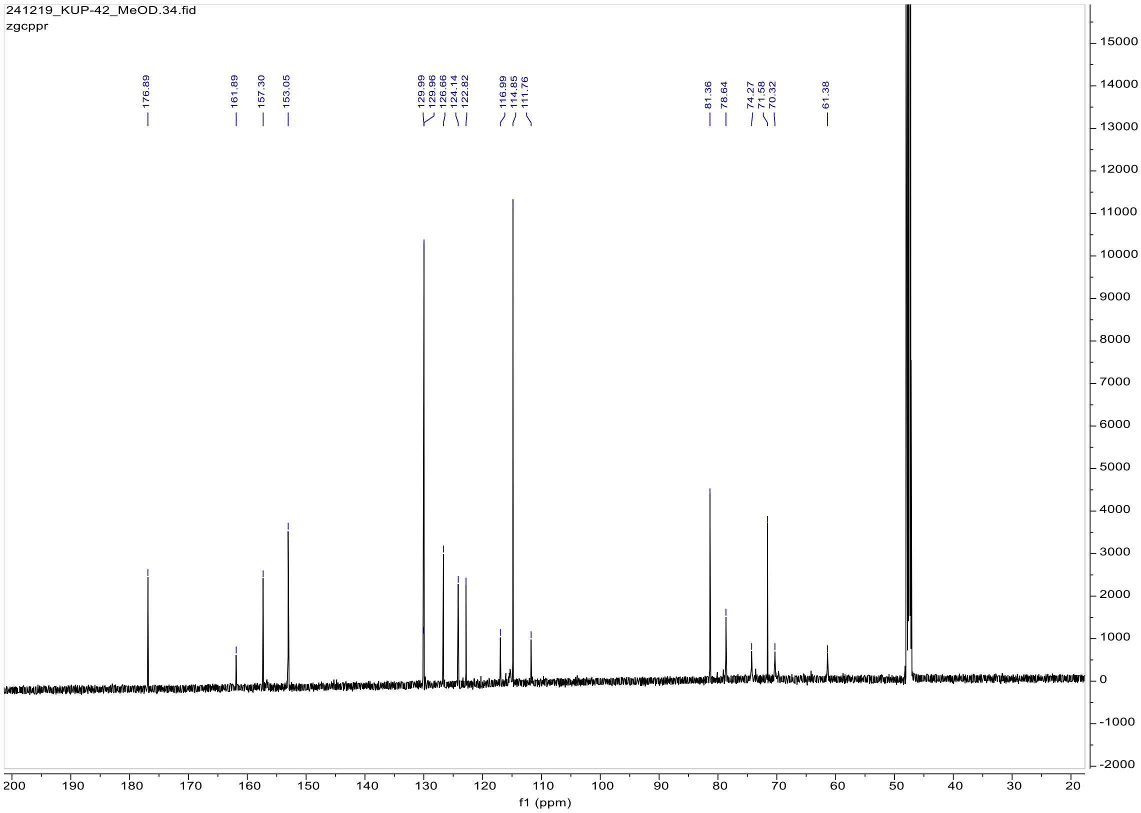

Figure S4. ^{13}C–NMR spectrum (150 MHz, CD_3OD) of puerarin isolated from *Pueraria lobata.*

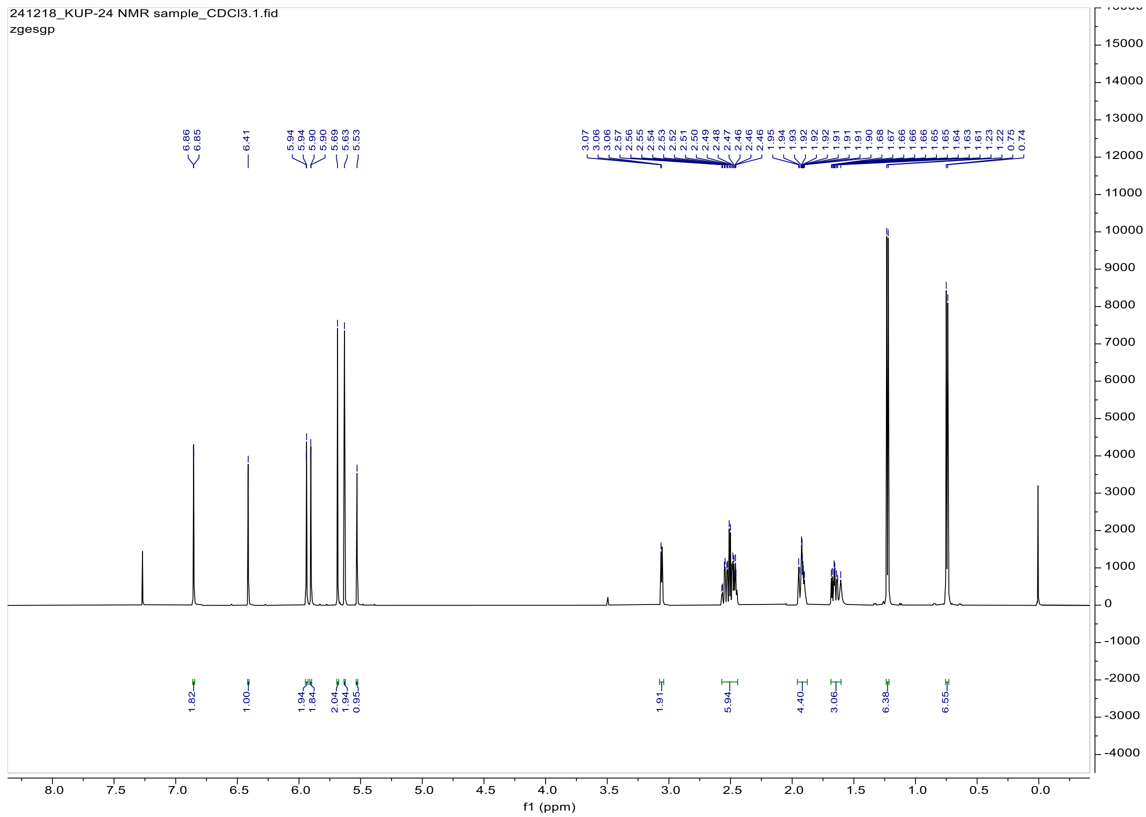

Figure S5. ^{1}H–NMR spectrum (600 MHz, $CDCl_3$) of sauchinone isolated from *Saururus chinensis*.

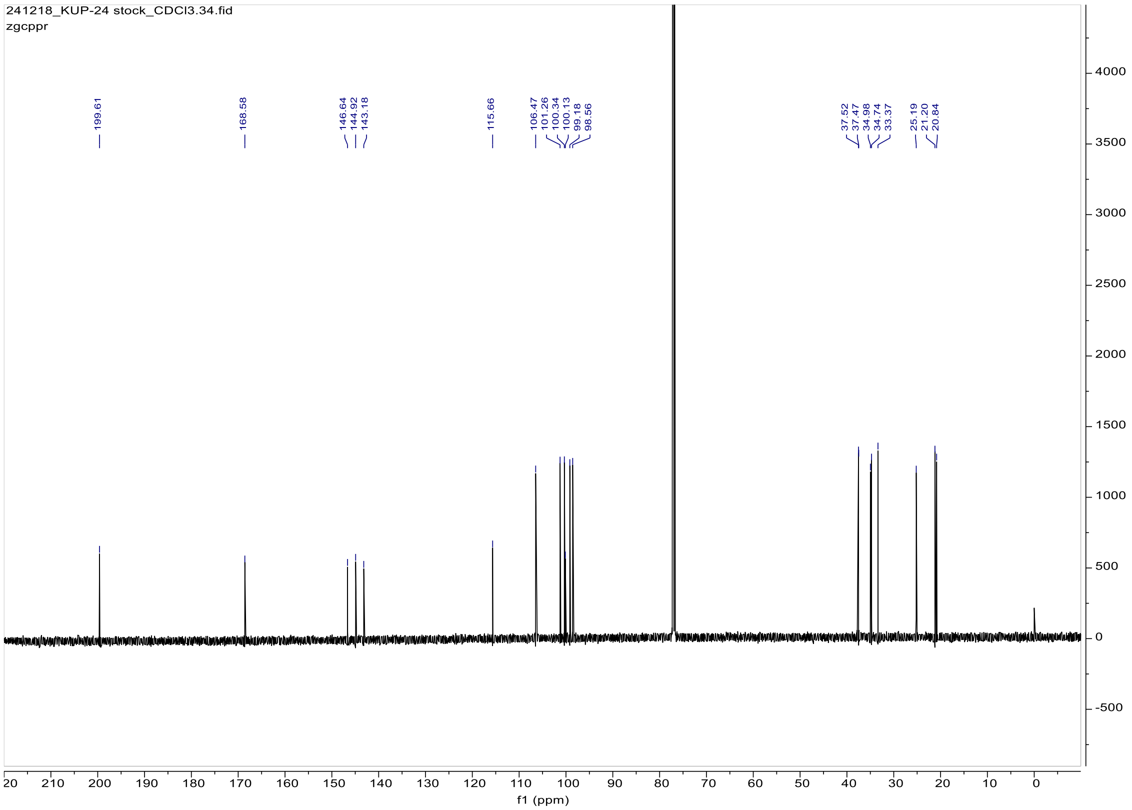

Figure S6. ^{13}C–NMR spectrum (150 MHz, $CDCl_3$) of sauchinone isolated from *Saururus chinensis*.

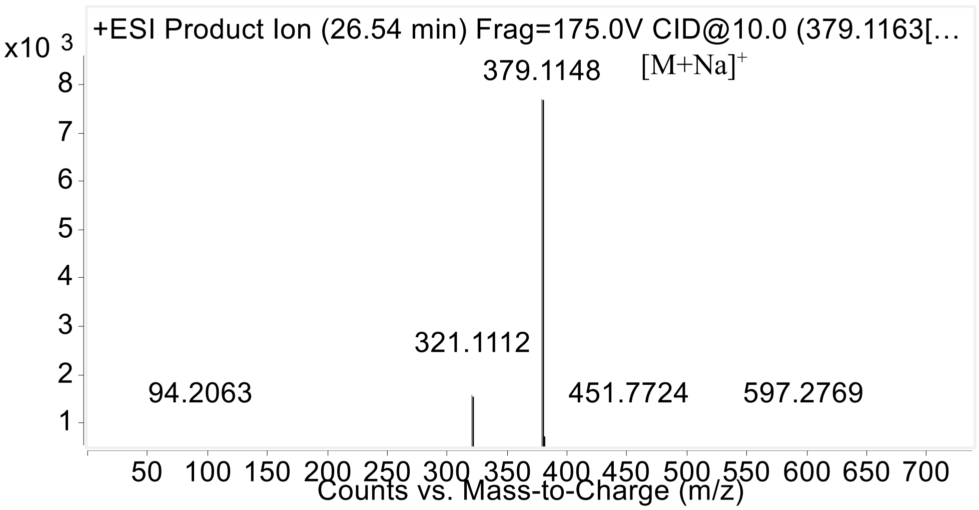

Figure S7. Electrospray ionization mass spectrometry of sauchinone.

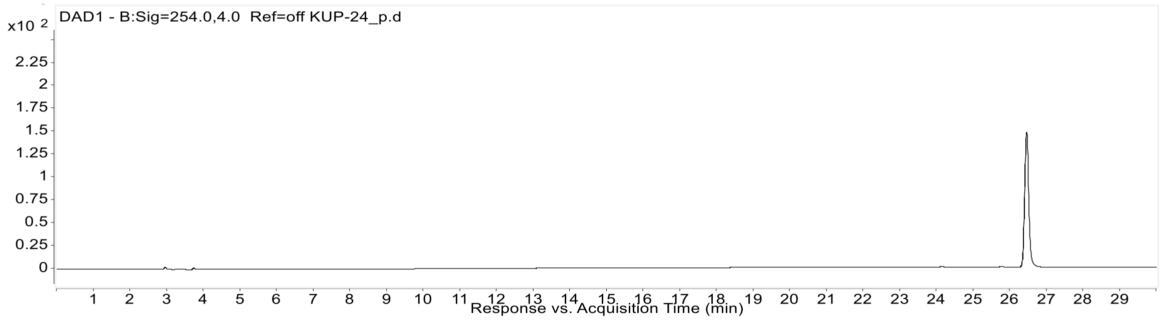

Figure S8. High-performance liquid chromatography chromatogram of sauchinone for purity verification. The UV chromatogram of sauchinone was acquired at 254 nm using Agilent Technology 1260 Infinity DAD. The purity was shown to be greater than 99 % when the area of peaks was integrated.
